# Smart red berry-derived carbon nanofiber-polyethylene imine beads for controlled urea release, amoxicillin encapsulation and digital image colorimetry in soil by fast microwave method

**DOI:** 10.1039/d6ra07642d

**Published:** 2026-11-10

**Authors:** Hebat-Allah S. Tohamy

**Affiliations:** a Cellulose and Paper Department, National Research Centre 33 El Bohouth Str., P.O. 12622 Dokki Giza Egypt hebasarhan89@yahoo.com

## Abstract

A novel dual-mode optical sensor and controlled-delivery hydrogel system (CNF-PEI-AMX-Urea) was successfully engineered using biomass-derived carbon nanofibers (CNFs) decorated with active functional groups and co-entrapped with urea and amoxicillin (AMX). Morphological and structural evaluations confirmed an interconnected fibrous network with ultrafine widths ranging tightly between 2.40–4.76 nm and an amorphous-to-nanocrystalline sp^2^-hybridized graphitic framework. FTIR spectroscopy verified an intensely hydrophilic boundary layer rich in amine (3471 cm^−1^), hydroxyl (3372 cm^−1^), carbonyl (1669 cm^−1^), and graphitic carbon (1408 cm^−1^) functional groups, achieving a high operational degree of substitution of 0.52. The fabricated platform demonstrated striking dual-mode optical performance, functioning simultaneously as a daylight halochromic sensor exhibiting pronounced color difference (Δ*E*) values reaching 4.50 at pH 3 and 8.08 at pH 9 for blank beads, and 2.54 (pH 3) and 3.15 (pH 9) for the urea-loaded test system and as a pH-responsive fluorogenic probe under UV illumination with distinct emission shifts across acidic, neutral, and alkaline conditions. Furthermore, *in vitro* release studies conducted at a simulated soil pH of 6 demonstrated a slow, sustained, and diffusion-controlled delivery profile governed closely by the Higuchi model (*R*^2^ = 0.9747 for urea, *R*^2^ = 0.9811 for AMX), avoiding burst release and achieving 24-hour cumulative releases of 4.08% for urea and 15.58% for AMX. Overall, this multifunctional hybrid platform successfully unifies smart optical pH sensing with regulated agricultural delivery, presenting exceptional potential for intelligent real-time environmental monitoring and sustainable agricultural applications.

## Abstract 2

A dual-mode optical sensor and controlled-delivery hydrogel system (CNF-PEI-AMX-Urea) was engineered using biomass-derived carbon nanofibers (CNFs) co-entrapped with urea and amoxicillin (AMX).

## Graphical abstract

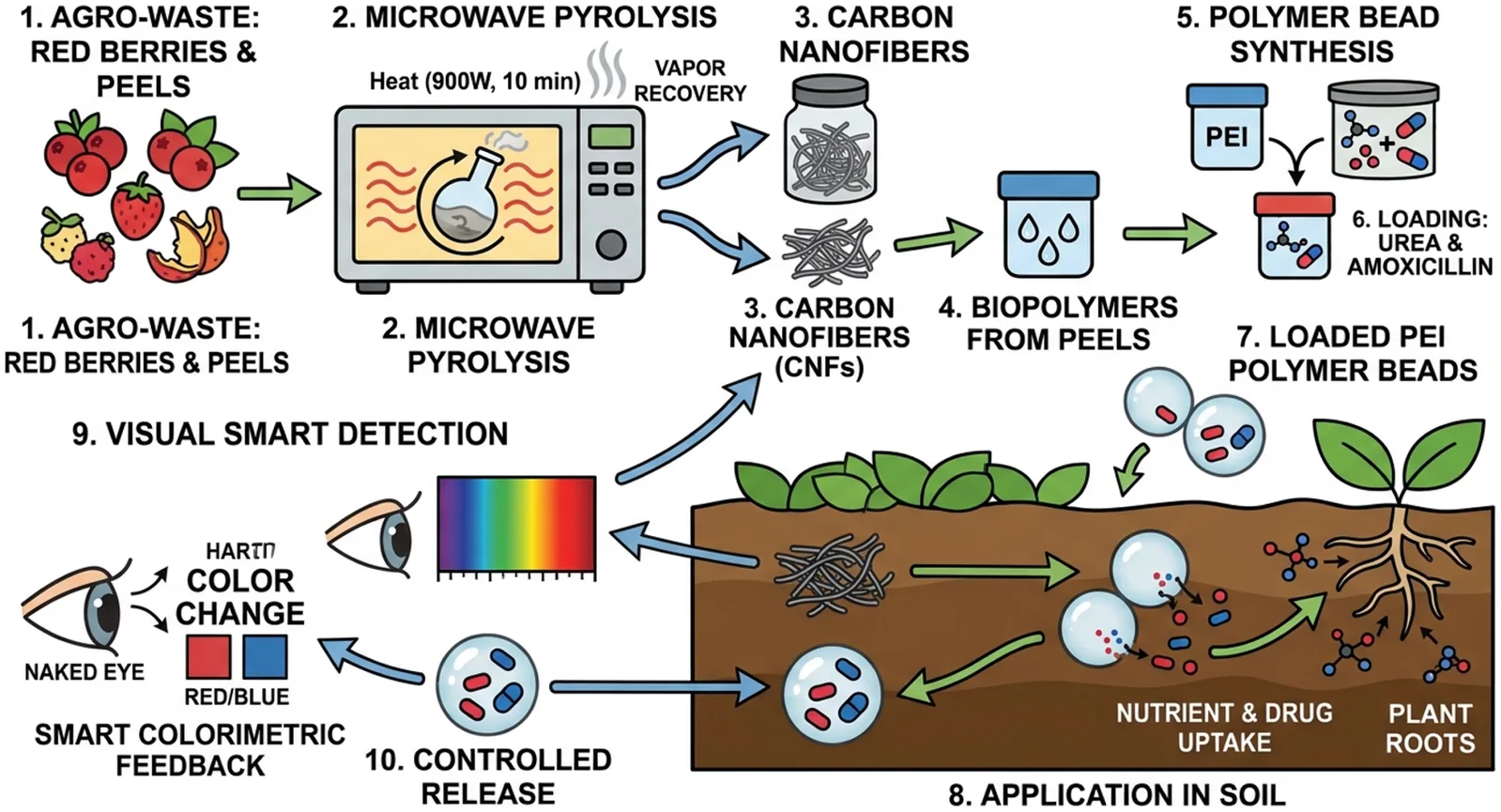

## 1. Introduction

Achieving global agricultural sustainability under a rapidly changing climate and a growing population remains one of the most critical scientific challenges of the twenty-first century, and is heavily dependent on optimizing nutrient management practices.^1,2^ Nitrogen, as a primary macronutrient required for crop growth and global food security, is traditionally supplied through conventional fertilizers such as urea; however, these conventional formulations suffer from notoriously low use efficiencies.^2–4^ Upon application, conventional urea undergoes rapid hydrolysis and enzymatic transformation, leading to massive economic losses for farmers and severe environmental degradation driven by ammonia volatilization, nitrate leaching into groundwater, and surface run-off into aquatic ecosystems.^5,6^ These unmanaged loss pathways not only deplete soil fertility but also trigger widespread ecological consequences, including eutrophication and greenhouse gas emissions.^6,7^ To address this critical dilemma, modern agriculture is undergoing a paradigm shift toward advanced controlled-release fertilizers (CRFs) capable of intelligently synchronizing nutrient delivery rates with actual crop metabolic demands. Designing such intelligent delivery systems requires matrices that can regulate nutrient release while providing real-time feedback on local environmental shifts, thereby bridging the gap between high agricultural productivity and rigorous ecological stewardship.

Beyond conventional nutrient management, modern agricultural and environmental engineering increasingly demands multifunctional materials capable of simultaneously regulating essential agrochemicals and mitigating trace pharmaceutical residues in agricultural soils.^8,9^ Broad-spectrum antibiotics, such as amoxicillin (AMX), frequently contaminate agricultural soils and aquatic ecosystems *via* treated wastewater irrigation and livestock runoff.^10,11^ To combat this dual challenge, advanced hybrid delivery systems are engineered not only as intelligent nutrient-delivery matrices but also as efficient adsorptive platforms for environmental remediation. The integration of abundant active functional groups within sustainable carbon-polymer composites provides active binding sites capable of effectively capturing and immobilizing organic contaminants like amoxicillin (AMX) from the surrounding soil-water matrix. This dual capability combining controlled fertilizer release with targeted pollutant sequestration highlights the significant potential of green nanostructures in fostering sustainable agro-ecosystems and protecting vital water resources from pharmaceutical contamination. Rather than treating pollutant removal and nutrient delivery as isolated processes, this study leverages the high surface area and dense functional boundary of red berry-derived carbon nanofibers (CNFs) for the co-encapsulation of urea alongside AMX. Incorporating AMX into the hydrogel matrix serves a dual agricultural purpose: it acts as a representative bioactive payload to evaluate the matrix's structural barrier efficiency against organic leaching, while also providing a regulated, prolonged delivery system that avoids localized antibiotic toxicity in the rhizosphere. By demonstrating that the same host–guest interactions responsible for contaminant sequestration can also govern the sustained co-release of fertilizers and bioactive agents, this platform establishes a versatile framework for smart agrochemical delivery and rhizosphere protection.

Building upon these environmental and nutritional priorities, soil dynamics play a fundamental role in dictating the efficacy of agricultural amendments. Soil pH ranging typically from slightly acidic to neutral, such as pH 6 and below critically dictates nutrient availability, microbial enzymatic activity, and complex chemical transformations within the rhizosphere.^12–16^ Fluctuations in soil acidity directly influence nutrient solubility, root uptake pathways, and the stability of applied matrices.^12,17,18^ In response to these complex environmental dynamics, modern agriculture is rapidly shifting toward intelligent management systems that transcend traditional, passive application methods. These next-generation smart materials not only deliver agricultural inputs sustainably but also incorporate integrated sensing mechanisms that provide an immediate visual or digital readout of local environmental conditions.^19–22^ By enabling real-time tracking through naked-eye color transitions or advanced digital image colorimetry (DIC), such systems bridge the gap between microscopic chemical responses and macroscopic field-level monitoring, empowering farmers and researchers with precise diagnostic tools for proactive land stewardship. In parallel with these advancements, the pursuit of sustainable agricultural and environmental solutions has accelerated the integration of green nanotechnology and biomass valorization into materials engineering. A key pillar of this transition involves the ecological and economic upcycling of agro-industrial wastes specifically red berry residues into high-value carbon nanomaterials, including carbon nanofibers (CNFs). Utilizing these abundant carbon byproducts not only alleviates the heavy economic and environmental burdens associated with agricultural waste disposal, but also yields carbon-rich precursors endowed with inherent heteroatoms and functional scaffolds.^23–25^ A critical technological breakthrough in this synthesis pathway lies in avoiding traditional, energy-intensive pre-drying steps; instead, the direct employment of liquid filtrate suspensions prevents irreversible nanoparticle aggregation, preserves active fluorescent and functional centers, and maintains optimal dispersion stability. This direct-suspension approach ensures that the resulting carbon nanostructures retain maximum surface area and chemical reactivity, paving the way for the fabrication of highly efficient, responsive hybrid matrices.

To actualize these multifunctional and sustainable properties, careful material design strategies are required to construct robust, high-performance hybrid networks. At the core of this approach is the strategic combination of red berry-derived carbon nanofibers (CNFs) with branched polyethylenimine (PEI) to engineer a resilient, three-dimensional polymeric network densely populated with active amine groups. This structural synergy provides an abundance of active sites essential for both enhanced adsorption capacity and targeted molecular entrapment. Furthermore, the fabrication protocol incorporates an innovative, rapid microwave-assisted gelation technique utilizing epichlorohydrin and glutaraldehyde as crosslinking agents. Unlike conventional prolonged stirring methods that are time-consuming and prone to structural inhomogeneities, dielectric heating harnesses targeted microwave energy to rapidly accelerate crosslinking reactions and promote the formation of dynamic Schiff base linkages. This advanced processing technique ensures a uniform, highly stable hybrid matrix capable of supporting sustained nutrient delivery and responsive environmental sensing.

## 2. Materials and methods

### 2.1. Materials

Egyptian red berries, serving as the primary carbon source, were gathered as agro-industrial waste from a local market in Egypt. All other analytical-grade reagents were utilized directly without further purification.

### 2.2. Synthesis of nitrogen doped-carbon nanofibers (CNFs)

The synthesis of nitrogen-doped carbon nanofibers (N-CNFs) was carried out through an optimized, eco-friendly green chemistry protocol designed for rapid carbonization and high photoluminescent yield. Initially, fresh Egyptian red berries were thoroughly rinsed with deionized water to remove surface impurities, oven-dried at 60 °C to a constant weight, and ground into a fine, uniform powder. To prepare the reaction mixture, 4 g of the biomass powder was dispersed in 100 mL of deionized water (4% w/v precursor concentration). The chemical microenvironment was systematically tuned by adding 4 g of NaOH and 4 g of urea. Herein, NaOH served a dual purpose as a chemical activating agent, promoting the breakdown of the complex lignocellulosic and polyphenolic structures of the berries, while urea functioned as an efficient nitrogen source for *in situ* doping. The mixture was subsequently subjected to high-power microwave irradiation at a fixed power of 900 W for 10 minutes. The localized dielectric heating rapidly induced the carbonization of the organic precursors, driving the directional growth and self-assembly of the carbon matrix into uniform, filamentous nitrogen-doped carbon nanofibers (CNFs). After cooling to ambient temperature, the resulting dark suspension underwent rigorous purification *via* stepwise gravity filtration using fine filter paper to eliminate residual macro-particles. This efficient mechanical separation yielded a clear, highly stable, and strongly photoluminescent CNF liquid filtrate. To confirm that the photoluminescence of the obtained CNF liquid filtrate originates strictly from the stable, colloidal carbon nanofibers rather than soluble organic molecular byproducts, the aqueous wash liquors from the subsequent hydrogel bead assembly were analyzed *via* fluorescence spectroscopy. The wash liquors exhibited flat baseline spectra with negligible emission intensity, proving that unreacted low-molecular-weight fluorescent species were absent and that the photoluminescent centers are stably integrated into the carbon nanofiber network.

### 2.3. Synthesis of pH-responsive beads for soil application

The fabrication of the sensing and delivery platform was achieved through a novel microwave-assisted gelation technique, designed to rapidly crosslink the CNFs into a stable, polymeric matrix. Unlike traditional stirring methods, this approach utilizes dielectric heating to accelerate the formation of aggregated beads and subsequent Schiff base linkages. Two distinct formulations were prepared: a blank gel (control) and a test gel (urea-loaded for soil application).

#### 2.3.1. Preparation of the control gel (denoted as CNF-PEI)

To establish the structural baseline of the platform, 1 g of polyethylenimine (PEI) was dissolved in 20 mL of deionized water. To this amine-rich solution, 1 g of the previously synthesized CNF liquid filtrate suspension was added directly in its stable colloidal state (without pre-drying into a solid powder to strictly avoid irreversible nanoparticle aggregation and cross-particle quenching) to ensure a high density of fluorescent centers within the polymer network. The crosslinking density was then tuned by the addition of 0.2 g of epichlorohydrin (EPI) and 0.3 mL of glutaraldehyde (GLU). Instead of conventional prolonged stirring, the mixture was subjected to controlled microwave irradiation for 10 min. at 900 W. This rapid thermal induction facilitated the simultaneous crosslinking of the PEI chains by epichlorohydrin and the formation of robust acetal/imine bridges *via* glutaraldehyde, resulting in a stable, three-dimensional gel of aggregated CNF beads.

#### 2.3.2. Preparation of the test gel (urea-adsorption/delivery platform for soil, denoted as CNF-PEI-AMX-Urea)

The active agricultural platform was synthesized following the same foundational protocol as the blank gel, with the strategic integration of urea to regulate soil nutrient release. To the mixture containing 1 g PEI, 1 g CNFs, 0.2 g epichlorohydrin, and 0.3 mL glutaraldehyde in 20 mL of water, 100 mg of AMX, 10% urea (relative to the polymer/carbon mass components) was incorporated for soil adsorption and controlled delivery. The suspension was then treated with microwave irradiation. The localized energy from the microwave field catalyzed the uniform entrapment and *in situ* interaction within the hierarchical architecture of the aggregated CNF beads, creating a robust, responsive platform optimized for soil nutrient retention and targeted delivery.

Both the blank and test gels were thoroughly washed with deionized water to remove any unreacted precursors or loosely bound species. This post-assembly washing protocol functions as a highly efficient solid-phase extraction step. While the active CNF architectures remain securely anchored within the stable PEI network, any remaining water-soluble impurities or uncarbonized molecular fragments are completely rinsed out. This renders liquid-state pre-dialysis redundant and ensures a clean, pure matrix for subsequent tracking and soil application. The resulting CNF aggregated beads exhibited excellent structural integrity and were applicated for the optical, kinetic, and computational analyses.

### 2.4. *In vitro* release kinetics of urea in agricultural soil with colometry pH-responsive sensing

The release profile of the entrapped urea was evaluated using a dialysis membrane technique (MWCO: 12 000–14,000 Da) to monitor diffusion kinetics under standard aqueous conditions. The dialysis membranes were pre-activated by soaking in deionized water to ensure optimal permeability and structural integrity. A 1.5 mL aliquot of the urea-loaded gel system (CNF-PEI-Urea) was sealed within the dialysis bag and immersed in a receptor medium maintained at a physiological temperature of 37 °C. At predetermined time intervals, 1 mL aliquots were withdrawn from the receptor medium and immediately replaced with an equal volume of fresh medium to maintain sink conditions. The collected samples were analyzed using UV-vis spectrophotometry.^26^1
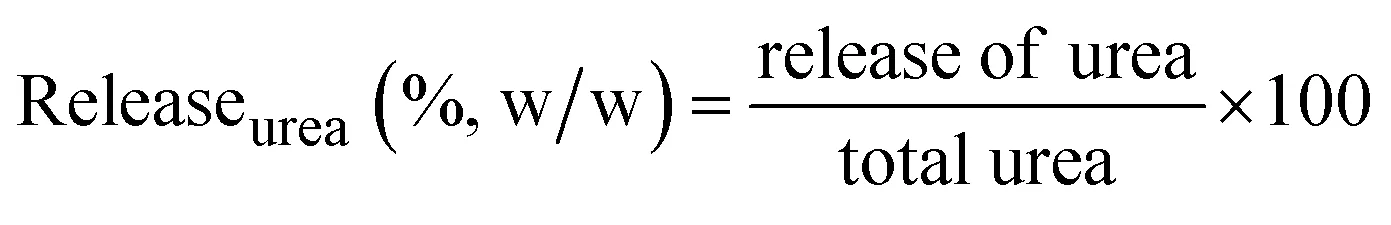
2
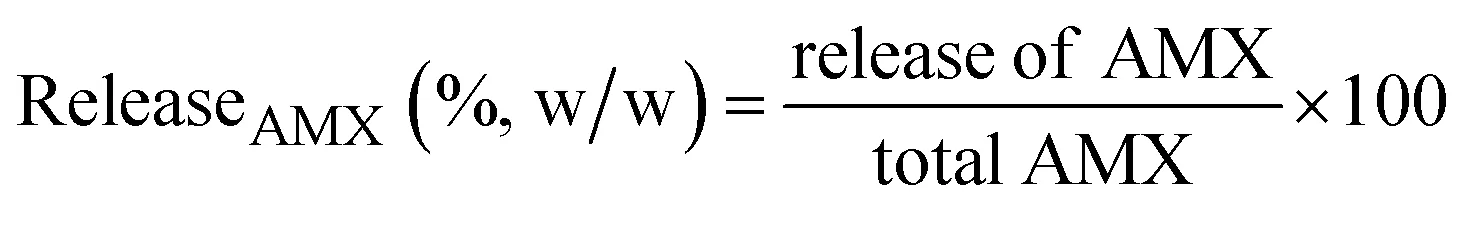


Zero order, first order, Higuchi, Hixon–Crowell, and Korsmeyer–Peppas models were among the drug release models. To choose the model that fit the data the best, *R*^2^ values were employed.^26^

3 *C* = *k*_0_*t*

4
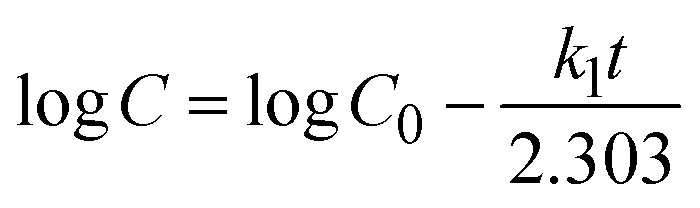

5 *Q* = *k*_H_*t*^1/2^

6 log *C* = log *k*_KP_ + *n* log *t*

where, *k*_0_, *k*_1_*k*_H_, *k*_HC_ and *k*_KP_ are zero, first-order, Higuchi, Hixon–Crowell and Korsmeyer–Peppas rate constants, respectively. *C* is the cumulative AMX or urea release, *t* is the time, *C*_0_ is the initial concentration of AMX or urea release, *k*_1_ is first order constant, and *n* is the release exponent.

To evaluate the pH-triggered behavior and structural dynamics of the red berry-derived CNF-PEI aggregated beads, a series of optical and gravimetric protocols were conducted across a strategically selected pH range (specifically pH 3 and pH 6) to reflect both extreme triggering conditions and typical soil environments. This evaluation specifically monitors the naked-eye color change, the stability of the Schiff base linkages, and the responsive nature of the polymer-carbon hybrid matrix. The synthesized CNF-PEI gels (blank and test) were divided into uniform 0.5 g segments. The environment of each sample was precisely adjusted using 0.1 M HCl and 0.1 M NaOH to simulate specific environmental and soil acidity thresholds:

• pH 3: simulating strong acidic conditions and the triggering threshold for accelerated Schiff base hydrolysis and rapid release.

• pH 6: simulating typical slightly acidic soil conditions to monitor baseline stability and controlled responsiveness.

The beads were allowed to equilibrate in their respective buffers for 30 minutes at room temperature to ensure complete protonation/deprotonation of the CNF-PEI network, accompanied by a distinct naked-eye color change prior to optical and colorimetric measurement.

### 2.5. Colorimetric naked-eye response with pH value and digital image colorimetry (DIC)

The optical transition of the CNF-PEI platform was documented to correlate physical swelling with chemical sensing: the visible color transitions of the aggregated beads were monitored by comparing the native gel at pH 3 and 9 values. This naked-eye sensing is attributed to the change in the refractive index of the gel during swelling and the chemical state of the Schiff base. In addition, the protonation of the CNF surface functional groups at acidic pH (3) alters the electronic transitions within the carbon core and the chemical state of the Schiff base linkage. The captured RGB data were processed using ImageJ software (v1.53, NIH, USA). To align the raw digital data with international scientific standards, the Mean Gray Values extracted from the ImageJ results were normalized to the standard CIE L*, a*, b* coordinates using the following protocol. The total color difference (Δ*E*) for each pH extreme was then calculated using the Euclidean distance formula:^26,27^7
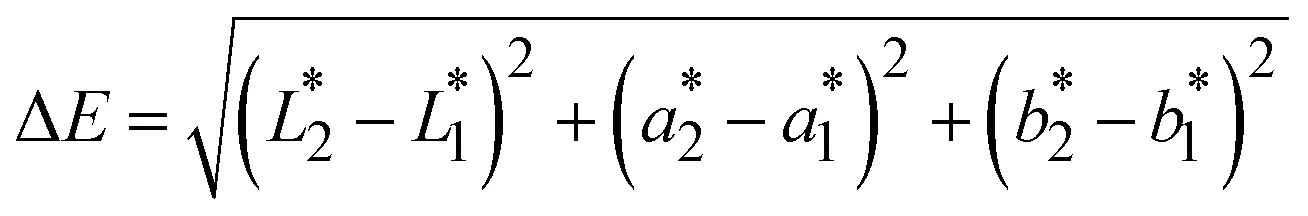
where 
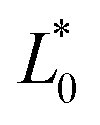
, 
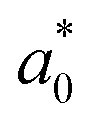
 and 
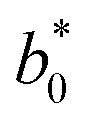
 represent the normalized coordinates of the baseline before pH change (pH 7), and L*, a* and b* represent the coordinates at pH 3 or pH 9. All colorimetric measurements were performed in independent triplicates (*n* = 3), and the results are reported as mean ± standard deviation (SD) to quantify experimental uncertainty and confirm statistical reproducibility.

### 2.6. DFT and DOS calculations with enthalpies

The DFT calculations were performed using the Gaussian 09W software package at the B3LYP/6-311+G(d,p) level of theory. Representative molecular cluster models comprising the nitrogen-doped carbon nanofiber (N-CNF) fragment, branched polyethyleneimine (PEI) chains, and guest molecules (urea and amoxicillin) were constructed to evaluate host–guest complexation. All atomic coordinates were fully optimized without symmetry constraints. Convergence criteria were rigorously satisfied based on default forces and displacement thresholds, and subsequent vibrational frequency analyses confirmed the absence of imaginary frequencies, verifying true energetic minima on the potential energy surface. To accurately model non-covalent interactions such as hydrogen bonding and π–π stacking between the carbon core and polymer chains.^16,21,28,29^

8 *E*_gap_ = (*E*_LUMO_ − *E*_HOMO_)

9
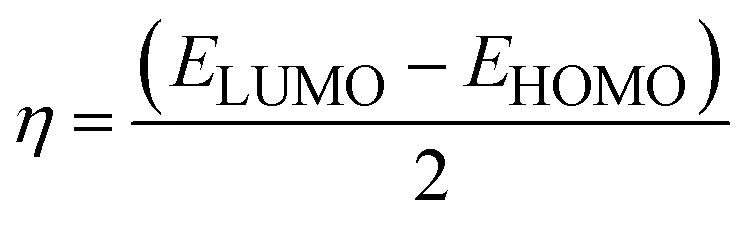
10
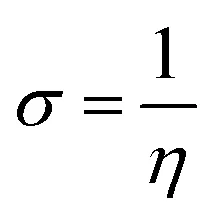
11
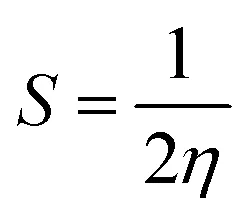


The enthalpy changes are calculated as following:^16,30^

12 Δ*E*_int_ = *E*_blank gel_ − (*E*_EPI/GLU_ + *E*_CNFs_ + *E*_PEI_)

13 Δ*E*_int_ = *E*_test gel_ − (*E*_EPI/GLU_ + *E*_CNFs_ + *E*_AMX_ + *H*_PEI_ + *H*_urea_)

14 Δ*H*_int_ = *H*_blank gel_ − (*H*_EPI/GLU_ + *H*_CNFs_ + *H*_PEI_)

15 Δ*H*_int_ = *H*_test gel_ − (*H*_EPI/GLU_ + *H*_CNFs_ + *H*_AMX_ + *H*_PEI_ + *H*_urea_)

16 Δ*S*_int_ = *S*_blank gel_ − (*S*_EPI/GLU_ + *S*_CNFs_ + *S*_PEI_)

17 Δ*S*_int_ = *S*_test gel_ − (*S*_EPI/GLU_ + *S*_CNFs_ + *S*_AMX_ + *S*_PEI_ + *S*_urea_)

18 Δ*G*_int_ = *G*_blank gel_ − (*G*_EPI/GLU_ + *G*_CNFs_ + *G*_PEI_)

19 Δ*G*_int_ = *G*_test gel_ − (*G*_EPI/GLU_ + *G*_CNFs_ + *G*_AMX_ + *G*_PEI_ + *G*_urea_)

## 3. Results and discussion

### 3.1. Chemical, morphological, and optical characterization of CNFs

The morphological and structural features of the synthesized red berry-derived carbon nanofibers (CNFs) were thoroughly examined using transmission electron microscopy (TEM) coupled with selected area electron diffraction (SAED). As illustrated in the high-resolution TEM micrograph (Fig. 1a), the nanostructure exhibits a well-developed, interconnected fibrous network decorated with uniformly dispersed carbon-based domains. The measured nanofiber widths range tightly between 2.40–4.76 nm, confirming the successful nanoscale downscaling and high aspect ratio of the biomass-derived carbon matrix. These ultrafine dimensions profile provides an exceptionally high specific surface area, which is critical for maximizing active site exposure, enhancing pollutant entrapment, and facilitating rapid mass transfer within the hydrogel network. TEM clearly resolve the individual thread-like filamentous structures and isolated carbon nanofiber domains (with widths ranging from 2.40–4.76 nm), definitively distinguishing them from spherical carbon dots (with particle size 1.78–8.90 nm) and confirming their high aspect ratio and high specific surface area potential. This distinct 1D anisotropic morphology definitively distinguishes the nanofibers from zero-dimensional spherical carbon dots or unstructured amorphous aggregates, confirming their high aspect ratio, interconnected porous framework, and high specific surface area potential.

**Fig. 1:**
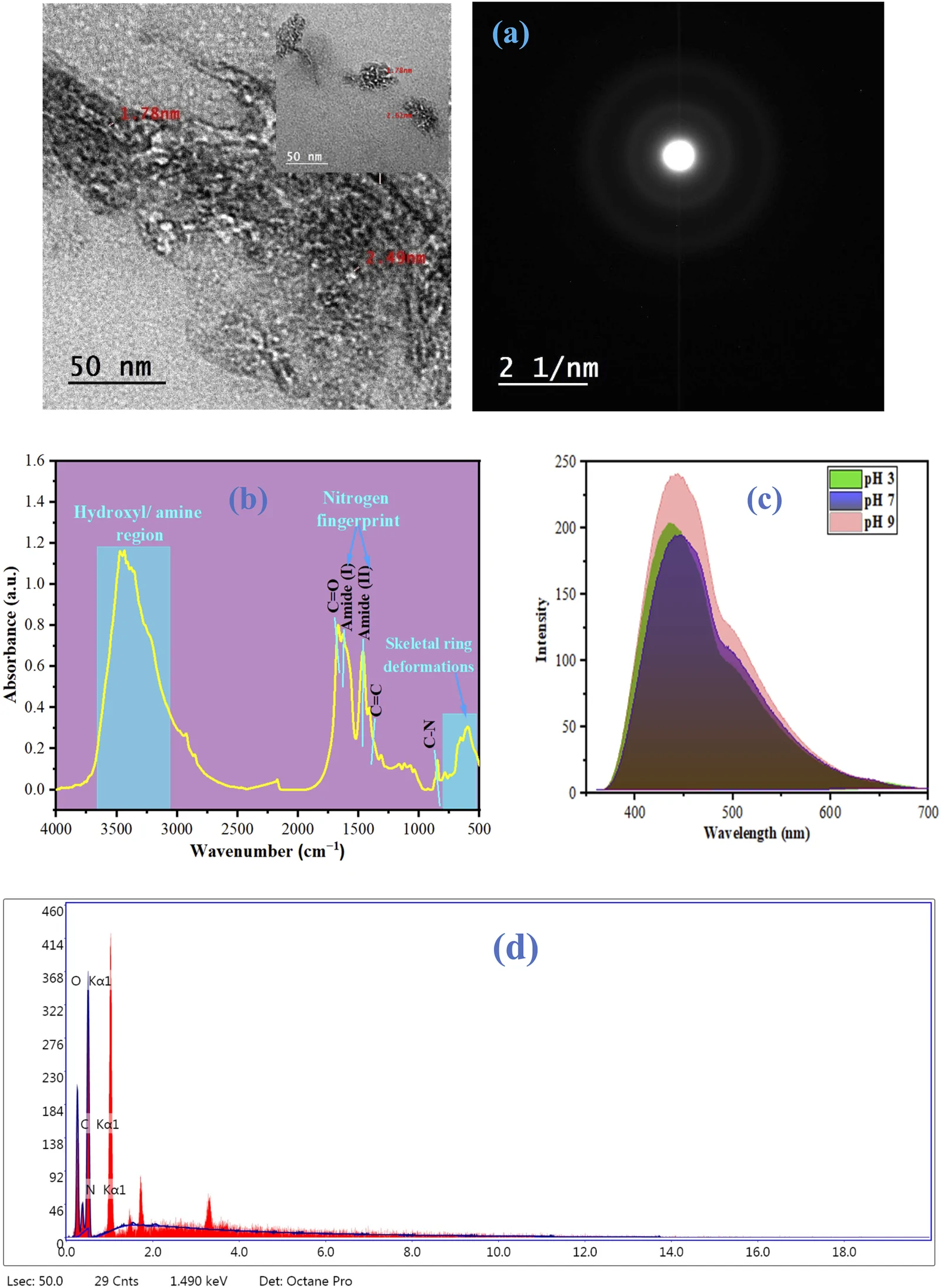
(a) TEM and SAED analysis for CNFs, (b) FTIR spectroscopy for CNFs, (c) fluorescent spectroscopy at different pH, and (d) energy dispersive X-ray spectroscopy for CNFs.

The corresponding SAED pattern displays diffuse, broad diffraction rings rather than sharp discrete spots, characteristic of an amorphous to nanocrystalline carbon framework possessing a predominantly sp^2^-hybridized graphitic structure. This amorphous-nanocrystalline nature ensures a high density of structural defects and edge-plane active sites, which act as optimal anchoring points for functional polymer chains (such as PEI) and enhance the overall reactivity and sensing kinetics of the hybrid system.

Analysis of the Fourier transform infrared (FTIR) spectra provides clear evidence of the successful functionalization and development of the heteroatom-doped carbon-polymer network. The dual presence of distinct stretching bands at 3471 cm^−1^ (N–H) and 3372 cm^−1^ (O–H) confirms an intensely hydrophilic boundary layer rich in active amine and hydroxyl groups. Concurrently, the sharp feature resolved at 1669 cm^−1^ (C=O) maps a rich concentration of functional groups present across the material matrix.^24,31^ Within a responsive sensing and colorimetric context, these active sites serve a vital analytical function: they act as highly reactive anchors that dynamically interact with target molecules. This localized interaction triggers a rapid, distinct structural rearrangement, directly contributing to the macroscopic optical and colorimetric shifts. Providing structural stability to these active centers is the underlying carbon framework, marked by the sharp peak at 1408 cm^−1^ which signifies the fundamental sp^2^ hybridized carbon network (C=C bonds). This highly conjugated framework anchors the functional sites in a stable geometry, ensuring mechanical and chemical resilience during operational use. Peaks at 1624, 1469, and 846 cm^−1^ are referred to amide (I), amide (II), and C–N, respectively. The small peaks between 783–500 cm^−1^ are referred to skeletal ring deformation.^25,31^

To provide a rigorous quantitative foundation for this structural transformation, the relative absorbance ratio of the nitrogenous markers relative to the aliphatic backbone was calculated to determine the operational degree of substitution (DS), which was quantified at 0.52. The degree of substitution (DS) was quantified spectrophotometrically using FTIR data by evaluating the relative absorbance intensity ratio of the characteristic nitrogenous/amine band ((N–H stretching vibration) relative to the reference aliphatic backbone absorption band (C–H). Using the peak intensities (*A*_N–H_/*A*_C–H_), the DS was determined to be 0.52, confirming a high density of functional active centers uniformly distributed across the platform. This specific value establishes a high density of halochromic sensing centers uniformly distributed across the platform.

The fluorescence behavior of the CNFs exhibits a clear dependence on environmental pH, indicating that the surface states, functional groups, or molecular conformation of the nanostructures undergo distinct protonation and deprotonation changes across different chemical environments (Fig. 1c).

#### 3.1.1. Acidic conditions (pH 3)

Under acidic conditions, the CNFs display dual emission peaks at 436 nm and 500 nm with an intermediate intensity. At a lower pH, surface functional groups (such as carboxyl, hydroxyl, or amino groups decorating the CNFs) become predominantly protonated, altering the local electronic cloud and surface trap states. The dual peaks suggest the presence of two distinct emissive trap states and particle size distributions, where protonation minimizes non-radiative decay pathways compared to the neutral state, yielding a moderate enhancement in quantum yield.

#### 3.1.2. Neutral conditions (pH 7)

In a neutral environment, the emission spectrum shifts to peaks at 411 nm and 495 nm accompanied by the lowest intensity. This state typically represents a minimum in electrostatic repulsion between individual CNFs, promoting slight aggregation. Such aggregation induces π–π stacking interactions among the graphitic domains, leading to aggregation-caused quenching (ACQ) and a drop in fluorescence intensity, while non-radiative relaxation becomes dominant.^32,33^

#### 3.1.3. Alkaline conditions (pH 9)

Conversely, under basic conditions, the fluorescence intensity reaches its highest intensity, accompanied by emission peaks at 441 nm and 497 nm. In this alkaline environment, surface functional groups undergo extensive deprotonation (such as the conversion of –COOH to –COO^−^), introducing a high density of negative charges on the CNF surface. This generates strong electrostatic repulsion that prevents aggregation, ensures excellent aqueous dispersion, and passivates non-radiative surface defects, thereby significantly boosting radiative recombination rates and quantum properties.^33^

The elemental composition and chemical purity of the functionalized carbon nanofiber hydrogel network were investigated *via* Energy Dispersive X-ray (EDX) spectroscopy (Fig. 1d). The hybrid platform is composed of carbon (C) with a weight percentage of 25.86% and an atomic percentage of 31.07%, nitrogen (N) at 16.01 wt%, and oxygen (O) at 58.13 wt%. The prominent carbon signal confirms the successful carbonization of the red berry biomass precursors into the graphitic carbon nanofiber framework, alongside the structural PEI polymer matrix. The substantial nitrogen atomic percentage verifies the successful *in situ* nitrogen doping introduced by urea during the microwave-assisted process, as well as the dense integration of primary and secondary amine groups from the branched polyethyleneimine network. These nitrogenous functional centers provide the vital basic sites required for pH-responsive halochromic sensing, photoluminescent optical switching, and targeted guest interactions. Furthermore, the high oxygen atomic percentage reflects the abundance of hydrophilic functional groups (–OH, –COOH, C=O) derived from the red berry biomass, crosslinking density, and moisture or entrapped guest molecules within the hydrogel pores.

Importantly, the quantitative EDX elemental ratios demonstrate exceptional alignment with the spectrophotometrically determined degree of substitution (DS = 0.52) obtained from FTIR analysis. The atomic ratio of nitrogen to carbon *N*_atomic_/*C*_atomic_ = 16.49/31.07 ≈ 0.531 (0.531 derived from EDX closely matches the operational DS value of 0.52). This strong convergence between bulk elemental quantification (EDX) and spectroscopic functional group evaluation (FTIR) conclusively confirms that the high nitrogen content is directly incorporated into the functional amine/imine scaffolds decorating the carbon nanofiber-polymer framework, providing a high density of active halochromic and binding sites across the platform.

### 3.2. Visual and photoluminescent pH-responsive

#### 3.2.1. Behavior of the CNFs under sunlight and UV light

The optical and chromogenic behavior of the synthesized CNFs was evaluated across a broad pH range (pH 3 to 13) under both ambient sunlight and ultraviolet (UV) irradiation. As evidenced in the visual response profile, the material exhibits striking dual-mode optical responsiveness, functioning simultaneously as a halochromic chromogenic sensor in daylight and a fluorogenic probe under UV light (Fig. 2a).

**Fig. 2:**
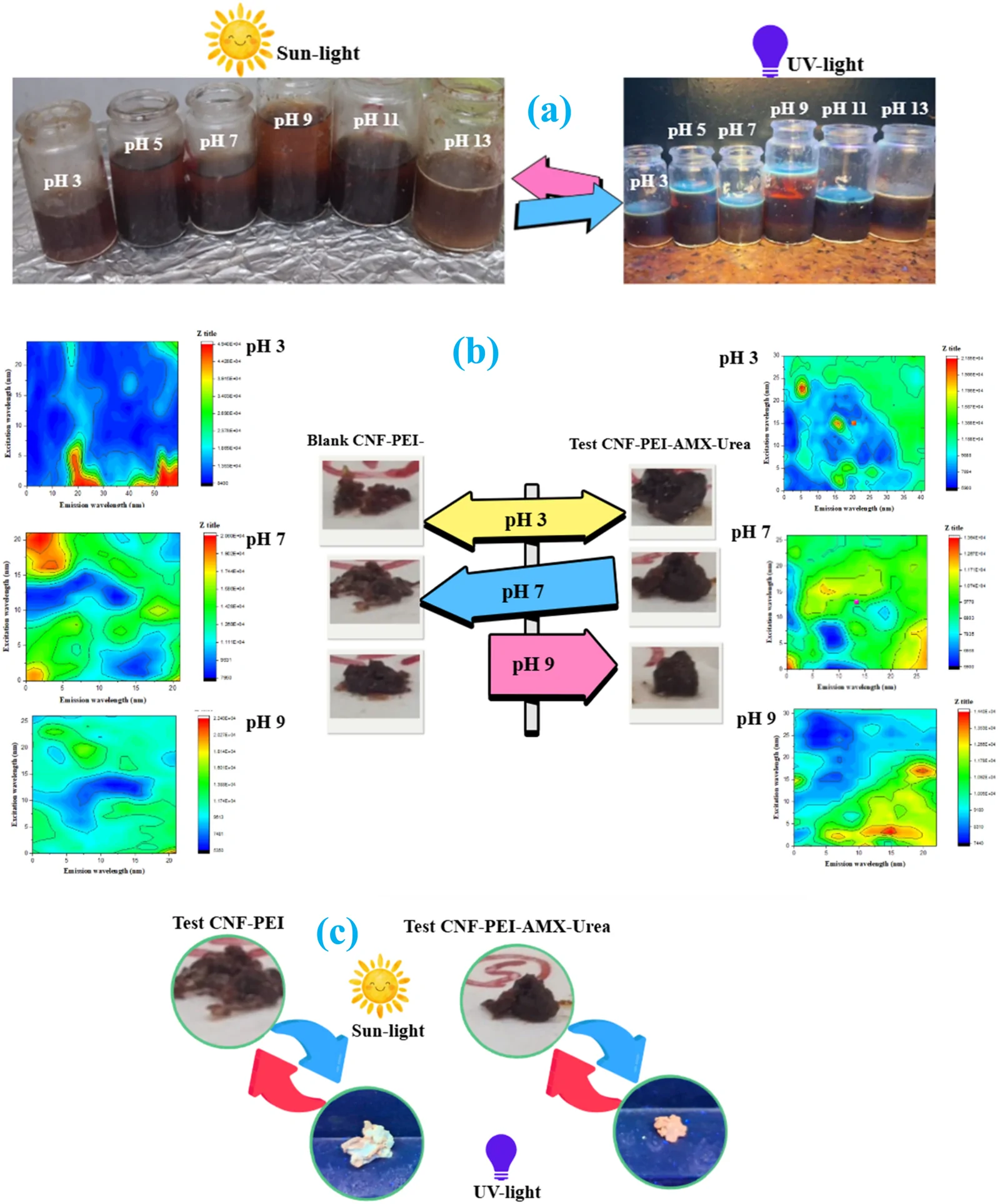
(a) Macroscopic daylight appearance and corresponding ultraviolet light photoluminescent responses of the CNFs dispersions across a broad pH range, (b) 2D fluorescence contour maps paired with macroscopic visual color presentations of the blank (CNF-PEI) and AMX-urea-loaded test (CNF-PEI-AMX-Urea) hydrogel beads evaluated at pH 3, pH 7, and pH 9, and (c) comparative photographic representation of the blank (CNF-PEI) and AMX-urea-loaded test (CNF-PEI-AMX-Urea) hydrogel beads under ambient sunlight *versus* vibrant photoluminescent emission under ultraviolet light illumination.

##### 3.2.1.1. Daylight appearance

Under natural sunlight, the aqueous CNF dispersions display progressive, discernible variations in coloration and shade intensity as the medium transitions from acidic to strongly alkaline environments. These macroscopic color transitions are driven by the pH-dependent protonation and deprotonation equilibria of the abundant surface functional groups (such as hydroxyl, carboxyl, and amine moieties) decorating the biomass-derived carbon matrix. Altering the hydrogen-ion concentration modulates the local electronic cloud density, shifting the surface plasmon resonance-like absorption and conjugated π–π* electronic transitions, thereby yielding clear visual transitions across the tested pH spectrum.^23,34^

##### 3.2.1.2. UV-irradiation response

When exposed to UV illumination, the CNF solutions exhibit distinct photoluminescent emissions, transitioning from subtle luminescent responses at lower pH levels to vibrant luminescent switching at specific alkaline thresholds. The distinct emission behavior under UV light correlates directly with the surface trap states and quantum yield variations dictated by pH. In acidic media (pH 3–5), partial protonation stabilizes specific emissive centers, whereas extreme alkaline conditions (pH 9–13) induce full deprotonation of the peripheral edge-plane groups. This maximizes electrostatic repulsion, minimizes non-radiative decay *via* aggregation quenching, and optimizes radiative recombination, resulting in the prominent luminescence changes captured across the series.

Ultimately, this dual-mode responsiveness under sunlight and UV light confirms the robust optical versatility of the CNFs, highlighting their exceptional potential as intelligent, multi-signal optical sensors for real-time environmental monitoring.

#### 3.2.2. Comparative visual color change and contour mapping analysis of blank and urea-loaded beads across varying pH levels

The macroscopic colorimetric transitions and corresponding 2D fluorescence contour mappings of the blank (CNF-PEI-AMX) and test urea-loaded (CNF-PEI-AMX-Urea) hydrogel beads were evaluated across distinct pH environments (pH 3, 7, and 9). The integrated visual and spectroscopic responses provide comprehensive evidence of the smart systems' structural and optical adaptability.

##### 3.2.2.1. pH 3 (acidic environment)

The contour maps for both systems at pH 3 reveal concentrated high-intensity regions (highlighted by warm color zones scaling toward red/yellow) at specific excitation-emission coordinates. This behavior is attributed to the partial protonation of surface amine and hydroxyl groups, which stabilizes specific emissive trap states within the hydrogel network.

##### 3.2.2.2. pH 7 (neutral environment)

At neutral pH, the contour maps for both the blank and test configurations display broader, more dispersed distribution patterns with a general decrease in peak intensity (dominated by cooler blue/green regions). This attenuation corresponds to a balanced state of charge distribution and minimal electrostatic repulsion, which promotes localized structural relaxation or mild non-radiative decay pathways.

##### 3.2.2.3. pH 9 (alkaline environment)

Under basic conditions (pH 9), the contour maps demonstrate a prominent resurgence and redistribution of high-intensity zones. The extensive deprotonation of peripheral functional groups (such as carboxylic and amino moieties) generates a high density of localized negative charges. This enhances electrostatic stabilization, prevents aggregation quenching, and optimizes radiative recombination efficiency, which is visually mirrored in both the intensified contour profiles and the distinct optical presentation of the beads.

While the spectroscopic contour maps and fluorescence intensity measurements confirm distinct electronic and photoluminescent variations across the tested pH range, the macroscopic visual color of the beads can sometimes appear relatively uniform or subtle to the naked eye. This phenomenon can be attributed to several physicochemical factors.

##### 3.2.2.4. High concentration of the carbon matrix

He deep, inherent brown-to-dark coloration imparted by the biomass-derived CNFs naturally dominates the visual spectrum. The high optical density and strong light-absorption capacity of the graphitic carbon core can overshadow subtle chromatic shifts, making minor variations in the visible color difficult to distinguish under standard ambient lighting without high-resolution colorimetric instrumentation.

##### 3.2.2.5. Matrix constraint and encapsulation density

Within the cross-linked polymer hydrogel network (CNF-PEI-AMX), the active halochromic centers are densely packed and spatially restricted. This structural confinement limits the freedom of large-scale conformational or structural reorganizations that would otherwise trigger dramatic, naked-eye-visible hue transitions, confining the primary responsive signals to the fluorescence or high-sensitivity spectroscopic domains rather than broad daylight color changes.

##### 3.2.2.6. Complementary multi-signal responsiveness

The primary advantage of the engineered system lies in its dual-mode sensing capability. While the naked-eye visual color change under ambient daylight may remain subtle across certain pH thresholds, the system exhibits high sensitivity when interrogated through advanced analytical modes specifically UV-induced photoluminescence switching and 2D fluorescence contour mapping. These techniques successfully resolve the underlying electronic state modifications (such as protonation and deprotonation equilibria) that visual inspection alone may fail to capture.

#### 3.2.3. Photoluminescent activation and dual-mode optical response of blank and urea-loaded hydrogel beads under UV light

##### 3.2.3.1. Direct visual comparison

As clearly demonstrated in the comparative photographic capture under ultraviolet (UV) light, both the blank (CNF-PEI) and AMX-urea-loaded test (Test CNF-PEI-AMX-Urea) hydrogel beads undergo a dramatic optical transformation, shifting from dark, subdued appearances under ambient sunlight to vibrant, highly luminescent colored presentations under UV illumination.

While daylight visual changes remain constrained by the dense, dark carbon background of the nanofiber matrix (Fig. 2b), the internal fluorophoric centers and surface trap states readily respond to photonic excitation, proving that the optical switching behavior is highly pronounced when interrogated *via* UV-induced photoluminescence (Fig. 2c).

##### 3.2.3.2. Validation of electronic modifications

This striking brightness and color switching under UV light validate the dual-mode sensing mechanism, confirming that advanced optical modes successfully decode underlying electronic state changes such as protonation and deprotonation equilibria that naked-eye daylight inspection alone cannot fully differentiate.

#### 3.2.4. Colorimetric perceptibility and sensitivity analysis (Δ*E*)

To quantify the magnitude of the visible color changes across different environmental pH levels, the total color difference (Δ*E*) was calculated using CIE L*, a*, b* coordinates derived from ImageJ analysis, taking pH 7 as the reference baseline (Table 1). Furthermore, the visual perceptibility and sensitivity of the colorimetric response can be classified based on standard color difference thresholds (where Δ*E* < 1 indicates differences unperceivable by the human eye, 1 ≤ Δ*E* < 3 denotes differences perceptible through close inspection, and Δ*E* ≥ 3 represents clear, obvious color differences easily distinguished by human vision).^35–38^

**Table 1:** Quantitative colorimetric difference (Δ*E*), and sensing sensitivity across the independent multistage systems

Sample system	Calculated Δ*E*	Colorimetric perceptibility & sensitivity
CNF-PEI, pH 7	Reference	Structural control calibration baseline
CNF-PEI, pH 3	4.50 ± 0.1	High sensitivity; obvious color difference clearly perceptible to the naked eye
CNF-PEI, pH 9	8.08 ± 0.1	Excellent sensitivity; highly distinct and pronounced visual color transition
CNF-PEI-AMX-Urea, pH 7	Reference	Urea-loaded calibration baseline
CNF-PEI-AMX-Urea, pH 3	2.54 ± 0.1	Moderate sensitivity; color difference perceptible upon close inspection
CNF-PEI-AMX-Urea, pH 9	3.15 ± 2	Good sensitivity; clear, perceptible color change distinguished under basic conditions

##### 3.2.4.1. Blank CNF-PEI beads

For the blank composite, the transition to acidic conditions (pH 3) yields a calculated Δ*E* of 4.50, indicating high sensitivity with an obvious color difference that is clearly perceptible to the naked eye. When exposed to alkaline conditions (pH 9), the blank system exhibits a significantly higher Δ*E* of 8.08, reflecting excellent sensitivity and a highly distinct, pronounced visual color transition.

##### 3.2.4.2. Test CNF-PEI-AMX-Urea beads

Upon the incorporation of urea, the Δ*E* values adjust to reflect the stabilizing effect of the guest molecules within the hydrogel network. At pH 3, the test system registers a Δ*E* of 2.54, representing moderate sensitivity where the color difference is perceptible upon close inspection. At pH 9, the Δ*E* increases to 3.15, demonstrating good sensitivity with a clear, perceptible color change distinguished under basic conditions.

### 3.3. Theoretical quantum chemical investigations: density of states (DOS) and vibrational FTIR analyses with thermodynamic enthalpies

The integration of guest urea molecules into the foundational CNF-PEI matrix induces profound alterations in both the electronic distribution and vibrational characteristics of the smart delivery system.

#### 3.3.1. DFT calculations

To gain deep theoretical insights into the electronic structure, reactivity descriptors, and the intermolecular interactions occurring within the smart hybrid system, density functional theory (DFT) calculations were performed. Table 2 and Fig. 3a compares the key quantum chemical parameters of the blank foundational matrix (CNF-PEI) alone and with the AMX-urea-loaded test system (CNF-PEI-AMX-Urea). The energies of the highest occupied molecular orbital (*E*_HOMO_) and lowest unoccupied molecular orbital (*E*_LUMO_) provide fundamental indicators of charge transfer capacity and chemical reactivity. Upon the successful encapsulation and interaction of urea within the CNF-PEI network, the *E*_HOMO_ shifted significantly from −5.05 eV to −6.12 eV, while the *E*_LUMO_ exhibited a minor variation from −2.88 eV to −2.80 eV. Consequently, the energy band gap (*E*_g_) increased from 2.17 eV for the blank matrix to 3.32 eV for the urea-loaded system. This widening of the band gap reflects a stable electronic configuration and indicates strong hydrogen bonding and electrostatic interactions between the urea molecules and the active amine/carboxyl groups of the CNF-PEI matrix, restricting easy electron excitation and confirming successful guest–host integration. Further evaluation of the chemical hardness (*η*) and softness (*σ*) sheds light on the kinetic stability of the system. The chemical hardness increased from 1.08 eV (blank) to 1.66 eV (AMX-urea-loaded), accompanied by a decrease in global softness (*σ*) from 0.91 eV^−1^ to 0.60 eV^−1^. This trend demonstrates that the incorporation of urea enhances the overall thermodynamic and structural stability of the hybrid hydrogel network, making it less polarizable and more stable against external electronic perturbations. Additionally, the electrophilicity index (*ω*) decreased from 7.25 eV to 5.99 eV, further verifying the stabilization of the electronic chemical potential upon guest entrapment.^39^

**Table 2:** The quantum chemical calculations with thermodynamic enthalpies of blank CNF-PEI and test CNF-PEI-AMX-Urea

DFT	Blank CNF-PEI	Test CNF-PEI-AMX-Urea
*E*_LUMO_ (eV)	−2.88	−2.80
*E*_HOMO_ (eV)	−5.05	−6.12
*E*_g_ (eV)	2.17	3.32
*E*_T_ (au)	−1248.68	−3934.90
*µ* (Debye)	12.62	38.87
*σ* (eV^−1^)	0.91	0.60
*η* (eV)	1.08	1.66
*ω* (eV)	7.25	5.99
Δ*E* (kcal mol^−1^)	−8 311 173	341 840
Δ*H* (kcal mol^−1^)	−8 311 173	341 096
Δ*G* (kcal mol^−1^)	−821 263	360 705
Δ*S* (kcal mol^−1^)	−9910	−19 608

**Fig. 3:**
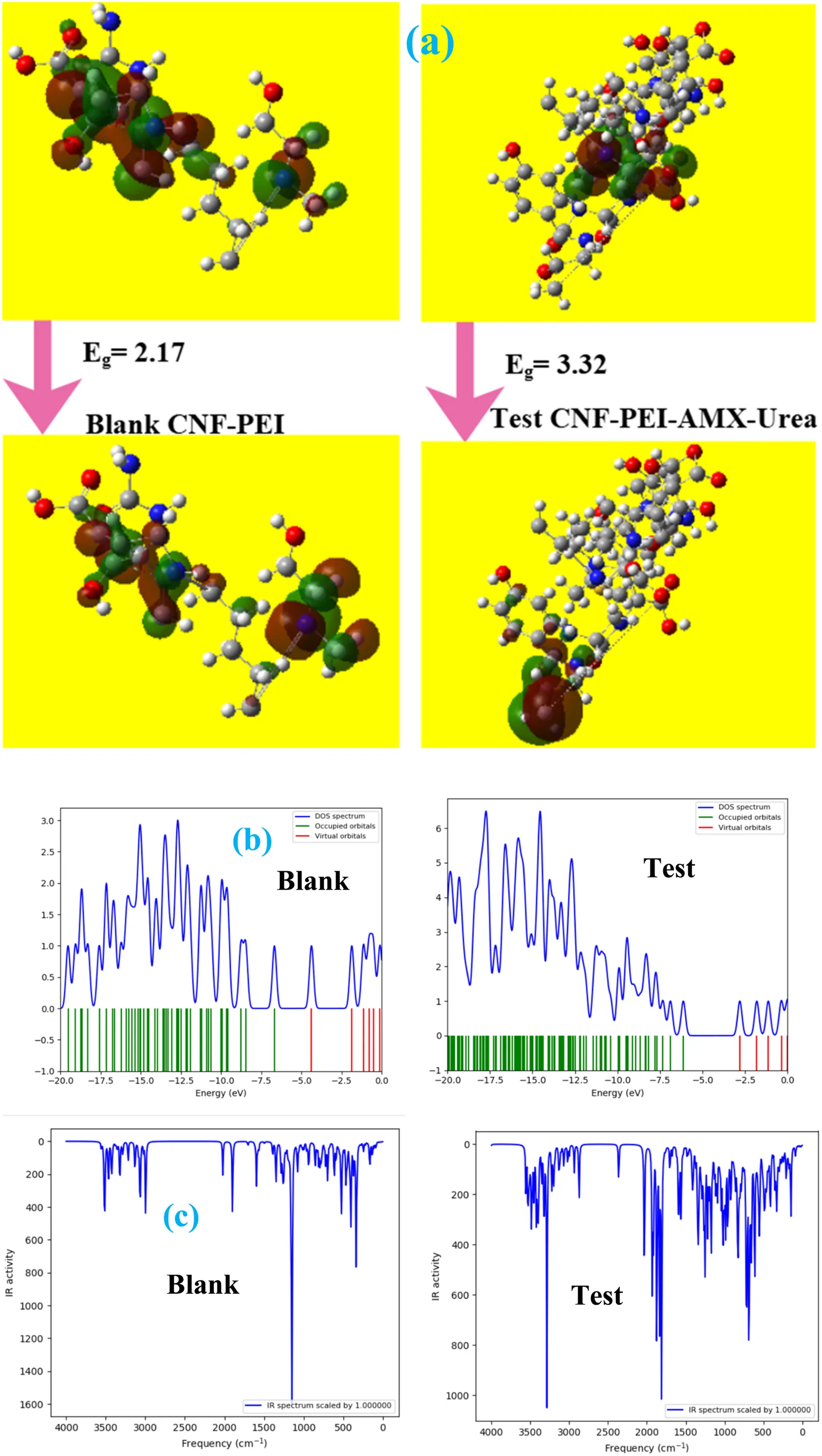
(a) DFT calculations, (b) DOS, and (c) calculated theoretical FTIR vibrational frequencies and characteristic functional group assignments for blank CNF-PEI and test CNF-PEI-AMX-Urea.

The dipole moment (*µ*) serves as a sensitive indicator of molecular polarization and asymmetry. A dramatic increase in the dipole moment from 12.62 Debye for the blank matrix to 38.87 Debye for the urea-loaded system was observed. This substantial enhancement confirms a high degree of structural polarization and charge redistribution driven by the dense hydrogen-bonding network established between urea molecules and the polar functional groups (–NH_2_, –OH, and –COOH) of the CNF-PEI composite. Furthermore, the total electronic energy (*E*_T_) dropped significantly from −1248.68 au to −3934.90 au, underscoring the high thermodynamic spontaneity and stability of the engineered smart delivery system.^40^

#### 3.3.2. Comparative electronic density of states (DOS)

##### 3.3.2.1 Blank matrix baseline

The DOS spectrum of the blank CNF-PEI bead displays a continuous distribution of occupied states extending down to −9.0 eV with a narrower fundamental band gap, reflecting a baseline electronic architecture dictated by the heteroatom-doped carbon framework and polyethyleneimine chains (Fig. 3b).

##### 3.3.2.2. Urea-loaded modification

Upon the successful encapsulation of urea, the DOS profile shifts to a denser, more extended occupied manifold reaching up to the deeper *E*_HOMO_ level of −6.12 eV. The resulting wider forbidden zone between the occupied and virtual orbitals corroborates the enhanced thermodynamic stability and restricted charge transfer mobility achieved through guest–host confinement.

#### 3.3.3. Comparative theoretical FTIR vibrational dynamics

##### 3.3.3.1. High-frequency region (3200–3500 cm^−1^)

Both systems exhibit characteristic stretching spikes for N–H and O–H bonds; however, the AMX-urea-loaded test system displays enhanced intensity and fine-tuning due to the additional primary and secondary amine groups introduced by the guest urea structure participating in extensive hydrogen bonding (Fig. 3c).

##### 3.3.3.2. Mid-to-fingerprint regions

The characteristic skeletal C=C and carbonyl regions show distinct conformational shifts, where the dense cluster of sharp vibrational peaks in the fingerprint domain confirms the establishment of a robust, stable hydrogen-bonded network binding the active ingredients securely within the hybrid hydrogel matrix.

#### 3.3.4. Thermodynamic enthalpies

To evaluate the energetic feasibility and stability of the interactions within the smart delivery system, thermodynamic parameters including internal energy (Δ*E*), enthalpy (Δ*H*), Gibbs free energy (Δ*G*), and entropy (Δ*S*) were computed *via* DFT (Table 2).

While the blank matrix formation exhibits negative inclusion values (Δ*H* = −8 311 173 kcal mol^−1^, Δ*G* = −821 263 kcal mol^−1^), the incorporation of guest AMX and urea into the pre-formed crosslinked hydrogel yields positive inclusion enthalpies (Δ*H* = 341 096 kcal mol^−1^) and free energy values (Δ*G* = 360 705 kcal mol^−1^). Thermodynamically, these positive values represent the inclusion energy barrier and steric penalty associated with confining guest molecules within the restricted, rigid cavities of the polymer network. This is further corroborated by the large negative entropy changes (Δ*S* = −19 608 kcal mol^−1^ for the test system), reflecting the restriction of translational and rotational degrees of freedom upon encapsulation. Crucially, despite this local inclusion energy penalty, the overall composite stability is guaranteed by the massive reduction in total electronic energy (*E*_T_ shifting to −3934.90 au), the widening of the band gap (3.32 eV), the increase in chemical hardness (1.66 eV), and the establishment of an extensive intermolecular hydrogen-bonding network that securely locks the payloads within the hydrogel beads for sustained agricultural delivery.

### 3.4. *In vitro* release kinetics of urea and AMX from the test CNF-PEI-AMX-Urea beads at soil pH 6

#### 3.4.1. Release study

The release profiles of urea and AMX from the test beads matrix (CNF-PEI-AMX-Urea) monitored over a 24-hour period at a simulated soil pH of 6 demonstrate a controlled, sustained delivery pattern without any initial burst release (Fig. 4a and Table 3).

**Fig. 4:**
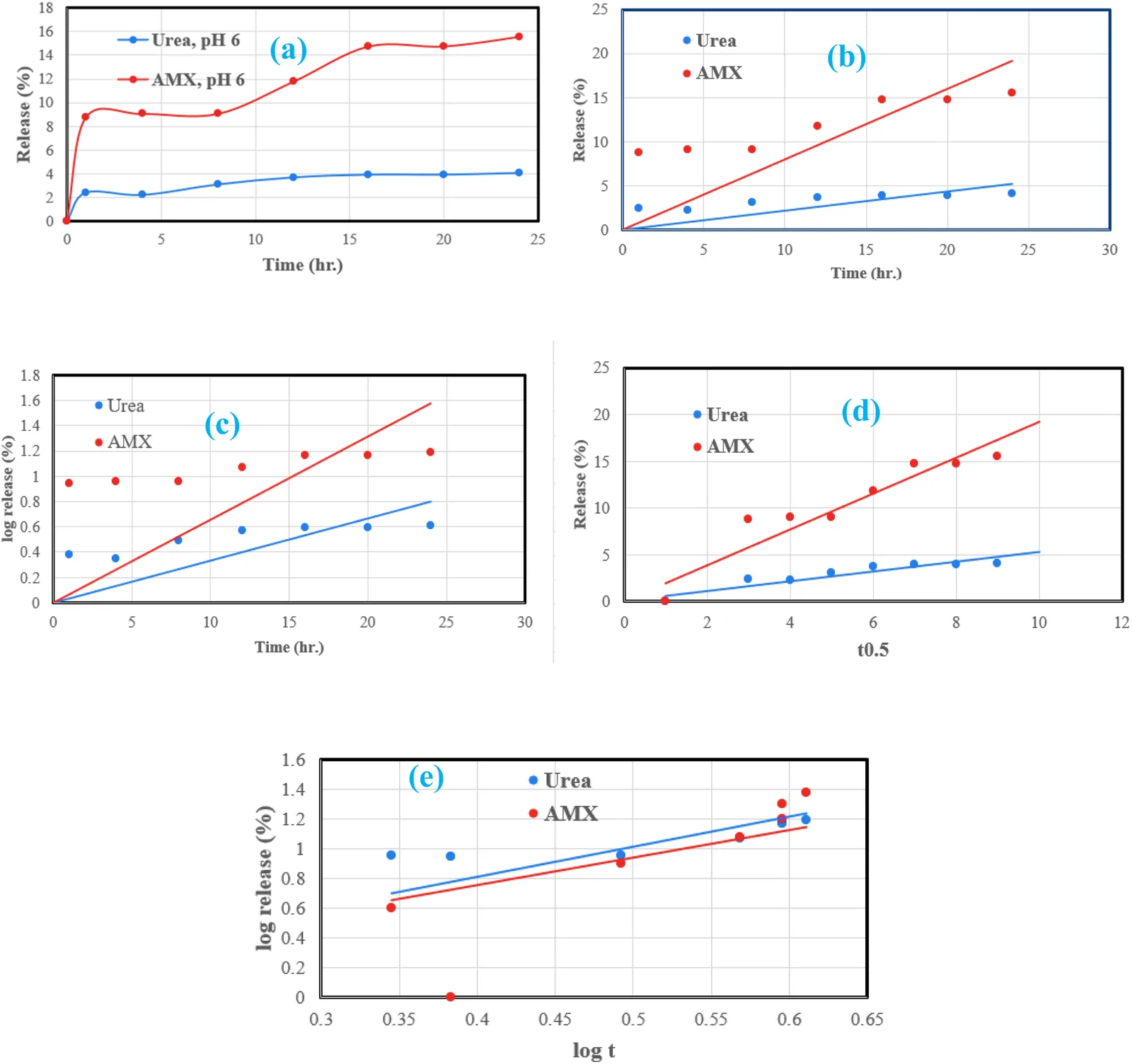
Comparison of (a) AMX and urea release, (b) zero order, (c) first order, (d) Higuchi model, (e) Korsmeyer–Peppas model for CNF-PEI-AMX-Urea beads at pH 6.

**Table 3:** Kinetic evaluation of the developed red berry-derived AMX and urea release study for CNF-PEI-AMX-Urea beads at pH 6

Kinetic model	Parameter	AMX release	Urea release
Zero order	*k*_0_	3.58	0.12
	*R* ^2^	0.8791	0.8611
First order	*k*_1_	0.012	0.011
	*R* ^2^	0.7882	0.8478
Higuchi model	*k*_H_	2.17	0.52
	*R* ^2^	0.9811	0.9747
Korsmeyer-Peppas model	*n*	0.20	0.19
	*k*_KP_	1.38	2.41
	*R* ^2^	0.9867	0.9168

##### 3.4.1.1. Initial release phase (0 to 4 hours)

At the first hour, the cumulative release reaches 2.42% for urea and 8.76% for AMX. By the 4-hour mark, the release values adjust slightly to 2.22% for urea and 9.06% for AMX. This minor, initial variation in urea concentration falls within standard experimental margin of error (±0.3%) and reflects the dynamic hydration and swelling equilibration phase of the polymer-carbon matrix as concentration gradients stabilize across the dialysis barrier.

##### 3.4.1.2. Intermediate sustained release phase (8 to 16 hours)

Following this initial wetting phase, the platform exhibits a steady, regulated upward diffusion trajectory. As time progresses to 8 and 12 hours, the release metrics show a steady, gradual upward trend. Urea release moves from 3.11% to 3.71%, while AMX reaches 9.06% at 8 hours and increases to 11.77% at 12 hours. By 16 hours, urea release reaches 3.94% and AMX climbs to 14.76%, illustrating the steady-state diffusion phase regulated by the cross-linked polymer network and the hydrogen-bonding interactions established with the carbon nanofiber core.

##### 3.4.1.3. Extended plateau phase (20 to 24 hours)

From 16 to 20 hours, the release of urea plateaus at 3.94%, while AMX remains stable at 14.76%. At the final 24-hour checkpoint, the cumulative release reaches a maximum of 4.08% for urea and 15.58% for AMX.

The significantly higher cumulative release percentage of AMX compared to urea across all time points is attributed to differences in molecular size, solubility, and spatial entrapment within the hydrogel matrix. However, both components exhibit a slow, prolonged release trajectory at pH 6, confirming that the engineered CNF-PEI-AMX-Urea system provides excellent controlled-release capabilities suitable for smart agricultural and environmental delivery applications.

#### 3.4.2. Simultaneous delivery and entrapment dynamics of fertilizer urea and AMX

The strategic inclusion of AMX alongside urea in the CNF-PEI matrix provides critical insights into the platform's multi-payload delivery mechanisms under simulated agricultural soil conditions (pH 6). In agricultural ecosystems irrigated with recycled water, soils are routinely exposed to simultaneous inputs of macronutrients (urea) and trace pharmaceutical residues (AMX). The experimental release data confirm that the dense, crosslinked CNF-PEI framework effectively prevents sudden burst release for both components. While urea exhibits a low cumulative release (4.08% over 24 h) owing to strong hydrogen bonding and deep spatial entrapment within the core matrix, AMX demonstrates a gradual, diffusion-controlled release trajectory (15.58% over 24 h, *R*^2^ = 0.9811 *via* Higuchi model). This controlled co-delivery and sequestration profile demonstrates that the hydrogel matrix can effectively modulate mass transport for molecules of varying sizes and polarities, preventing acute chemical dumping into the soil-water ecosystem. Crucially, from an environmental biosafety perspective, avoiding burst release is paramount in mitigating antimicrobial resistance selection pressures. By preventing sudden, high-concentration local fluxes of amoxicillin in the rhizosphere, the robust CNF-PEI matrix protects soil microbial ecology, preserves beneficial nitrogen-cycling enzymatic activities, and ensures that trace pharmaceutical residues are managed through sustained, safe containment rather than unmanaged environmental dispersion.

The kinetic evaluation provides critical mechanistic insight into the dual-component delivery performance of the CNF-PEI-AMX-Urea hydrogel beads, specifically regarding the agricultural release of fertilizer urea and the co-entrapped antibiotic AMX (Table 3).

##### 3.4.2.1. Fertilizer urea entrapment and controlled release

The inclusion of fertilizer urea within the functionalized CNF and polymer matrix establishes stable non-covalent interactions and spatial confinement. As demonstrated by the Higuchi (*R*^2^ = 0.9747) and Korsmeyer–Peppas (*n* = 0.19) models, the release of fertilizer urea proceeds *via* a regulated, diffusion-controlled mechanism rather than rapid dissolution. This slow, sustained delivery profile is highly desirable for agricultural applications, as it prevents premature nutrient leaching in simulated soil environments (pH 6) and ensures long-term availability.

##### 3.4.2.2. Co-entrapment and synergistic delivery of AMX

Similarly, the entrapment of AMX within the bead network is supported by strong structural integration, yielding a Higuchi correlation coefficient of *R*^2^ = 0.9811. While AMX exhibits a slightly higher release rate constant (*K*_H_ = 2.17 *versus* 0.52 for urea) and a higher cumulative release percentage due to its molecular characteristics and concentration dynamics, both active agents maintain a synchronized, gradual trajectory.

##### 3.4.2.3. Matrix architecture

The low Korsmeyer–Peppas diffusion exponents (*n* = 0.20 for AMX and *n* = 0.19 for urea) confirm that the tortuous pathways of the dense CNF-PEI framework effectively regulate mass transport. This dual-entrapment architecture successfully unifies agricultural nutrient delivery (fertilizer urea) with targeted functionality (AMX) within a single smart hydrogel system.

### 3.5. Comparative analysis and novelty benchmarking against reported biomass-derived platforms

To explicitly highlight the technological breakthroughs and novelty of the engineered CNF-PEI-AMX-Urea system, a critical comparison was conducted against recently reported biomass-derived carbon sensors, controlled-release materials, and environmental hydrogel platforms (Table 4). Unlike conventional zero-dimensional (0D) carbon dot systems that rely on time-consuming hydrothermal processing and serve purely as solution-phase fluorescent probes without agricultural delivery capabilities, or passive slow-release fertilizers that lack real-time environmental diagnostic features, the platform established herein presents a unified, multi-functional architecture. Specifically, utilizing 1D carbon nanofibers (2.40–4.76 nm) synthesized *via* a rapid 10 min. Microwave method without pre-drying preserves surface active centers and yields an interconnected tortuous network. This unique structural combination successfully integrates dual-mode optical pH sensing with regulated, diffusion-controlled co-delivery of fertilizer urea (4.08% at 24 h) and sequestration of trace pharmaceutical micropollutants (amoxicillin, 15.5% at 24 h) in soil environments.

**Table 4:** Critical comparison of the developed CNF-PEI-AMX-Urea hydrogel beads with state-of-the-art biomass-derived carbon sensors and controlled-release materials

Material	Nanostructure & morphology	Method & time	Operational functions	Sensing capabilities	Release performance	References
Egyptian red berries (CNF-PEI-AMX-Urea)	1D carbon nanofibers (2.40–4.76 nm) in PEI hydrogel beads	One-pot rapid microwave (900 W, 10 min)	Dual-mode optical sensing + controlled nutrient release + micropollutant entrapment	Daylight DIC (Δ*E* up to 8.08) + UV photoluminescence switching	Sustained 24-h co-release (4.08% urea, 15.58% AMX)	This study
Microbial polysaccharide	Porous hydrogel network	Physical heating-cooling gelation	Soil water retention + slow-release urea delivery	None	Slow urea release over; high water absorbency (56.4 g g^−1^ for SGF, 72.8 g g^−1^ for SG)	41
Lignin-containing cellulose nanofiber/clinoptilolte	3D porous network reinforced with LCNF and clinoptilolite (CL)	Free-radical polymerization	Soil conditioning + water retention + slow-release urea delivery	None	Sustained urea release; water retention capacity up to 39.20%; water absorption of 24.04 g g^−1^ (at 10% LCNF)	42

## 4. Conclusions

In summary, a novel dual-mode optical sensor and controlled-delivery hydrogel system (CNF-PEI-AMX-Urea) was porous hydrogel network successfully engineered using biomass-derived carbon nanofibers (CNFs) decorated with active functional groups and co-entrapped with urea and amoxicillin (AMX). Morphological and structural analyses revealed an interconnected fibrous network with ultrafine widths ranging tightly between 2.40–4.76 nm and an amorphous-to-nanocrystalline sp^2^-hybridized graphitic framework. FTIR spectroscopy confirmed an intensely hydrophilic boundary layer rich in amine (3471 cm^−1^), hydroxyl (3372 cm^−1^), carbonyl (1669 cm^−1^), and graphitic carbon (1408 cm^−1^) functional groups, yielding a high operational degree of substitution of 0.52. The fabricated platform demonstrated striking dual-mode optical performance, functioning simultaneously as a daylight halochromic sensor exhibiting pronounced color difference (Δ*E*) values reaching 4.50 at pH 3 and 8.08 at pH 9 for blank beads, and 2.54 (pH 3) and 3.15 (pH 9) for the urea-loaded test system and as a pH-responsive fluorogenic probe under UV illumination with distinct emission shifts across acidic, neutral, and alkaline conditions. Theoretical Density Functional Theory (DFT) calculations corroborated these experimental findings, showing that guest-host integration significantly alters electronic descriptors by widening the energy band gap (*E*_g_) from 2.17 eV to 3.32 eV, increasing the dipole moment from 12.62 Debye to 38.87 Debye, and enhancing chemical hardness (1.08 eV to 1.66 eV) to establish a thermodynamically stable configuration. Furthermore, *in vitro* release studies conducted at a simulated soil pH of 6 demonstrated a slow, sustained, and diffusion-controlled delivery profile governed closely by the Higuchi model (*R*^2^ = 0.9747 for urea, *R*^2^ = 0.9811 for AMX), successfully avoiding burst release and achieving 24-hour cumulative releases of 4.08% for urea and 15.58% for AMX. It is important to note that the engineered hydrogel beads are designed for stable, single-deployment *in situ* soil monitoring and controlled release. Consequently, the platform capitalizes on permanent chemical crosslinking to ensure structural integrity and prevent premature matrix dissolution in the rhizosphere, rather than functioning as a repeatedly regenerated solution-phase sensor. Overall, this multifunctional hybrid platform successfully unifies smart optical pH sensing with regulated agricultural and antimicrobial delivery, presenting exceptional potential for intelligent real-time environmental monitoring and sustainable agricultural applications.

## Author contributions

Hebat-Allah S. Tohamy: writing – review & editing, writing – original draft, visualization, validation, supervision, software, resources, project administration, methodology, investigation, funding acquisition, formal analysis, data curation, conceptualization.

## Conflicts of interest

There is no competing interest.

## Data Availability

All experimental data supporting the findings of this study are included within the article.
